# The Chocolate Curriculum: A Gateway to Materials Science and Engineering and Python Programming

**DOI:** 10.1021/acs.jchemed.5c01442

**Published:** 2026-04-15

**Authors:** Janine K. Nunes, Ananya Chakravarti, Z. Vivian Feng

**Affiliations:** † Princeton Materials Institute, 6740 Princeton University, Princeton, New Jersey 08544, United States; ‡ Mechanical and Aerospace Engineering, Princeton University, Princeton, New Jersey 08544, United States; § Chemical and Biological Engineering, Princeton University, Princeton, New Jersey 08544, United States; ∥ Center on Science and Technology, Princeton University, Princeton, New Jersey 08544, United States

**Keywords:** High School/Introductory Chemistry, Curriculum, Interdisciplinary/Multidisciplinary, Inquiry-Based/Discovery Learning, Hands-On Learning/Manipulatives, Computer-Based Learning Materials Science, Thermal Analysis

## Abstract

Most incoming college students have little awareness of materials science and engineering (MSE) as a discipline, resulting in barriers to entry for a field central to modern technology. To address this gap, we designed a short course curriculum that introduced high school students to MSE concepts through accessible, hands-on, and computationally driven activities framed in the context of chocolate science. The curriculum, rooted in the Context-Based Learning framework, aimed to build scientific literacy and present an engineering perspective by linking everyday observations to the underlying materials science tetrahedron of “structure, process, properties, and performance” relationships. Students engaged in low-cost activities that explicitly connected to advanced laboratory techniques, such as differential scanning calorimetry and polarized optical microscopy, introducing them to tools that produce research-quality data but rely on the same principles. Computational modules introducing Python for modeling, quantitative analysis, and data visualization showed how computational skills can aid broader research efforts and enabled students to produce publication-quality figures and investigate hypotheses. This integrated experimental–computational approach with the chocolate theme lowered barriers to MSE, increased awareness of the field, and empowered students to become citizen scientists.

## Graphical abstract

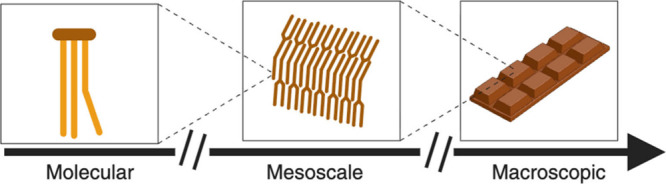

## Introduction

Materials science and engineering (MSE) plays a central role in developing the technologies that underpin modern society, yet most incoming college students have little exposure to the field or awareness of it as a distinct discipline. At the high school level, in particular, science instruction tends to emphasize established concepts in chemistry, biology, and physics, leaving students with limited opportunities to explore how these subjects integrate in the study of real-world materials. This lack of early exposure creates barriers to student engagement with MSE and to considering it as a future area of study.

Introduction to MSE is typically designed to expose students to the relationships among the structure, properties, processing, and performance (often known as the “material tetrahedron”)^1,2^ of a wide variety of materials—metals, ceramics, polymers, and composites. Curricula often begin with foundational concepts such as atomic bonding, crystal structures, and defects, which are then linked to macroscopic mechanical, thermal, and electric properties. To make these ideas concrete, curricula focused on polymers^1,3,4^ and metals^2^ are popular, offering familiar and tangible materials as entry points for precollege and entry-level college students. As an example, a recent study published in The Journal reports teaching materials science, in particular, polymer science, at “a soccer-themed science camp in Brazil,” highlighting the creativity in building pathways to materials science.^4^ Another popular entry point to MSE is to refocus an introductory chemistry curriculum on solid materials, including curriculum with an emphasis on novel nanomaterials.^5,6^ Although typical introductory chemistry courses center on small molecules, gases, and liquids, solids are essential to our materials world and offer rich examples to demonstrate fundamental chemistry concepts.

In this study, we report a summer short course curriculum for high school students focusing on the materials science of chocolate. The curriculum is grounded in the context-based learning (CBL) framework, centered around the idea of using real-world contexts and familiar materials to guide students’ learning.^7^ CBL has been shown to make abstract and unfamiliar concepts more meaningful and to improve students’ attitudes toward science, without compromising their understanding of scientific content when compared with conventional instructional approaches at the high school level.^8–10^ In addition, studies indicate that CBL can foster scientific creativity by encouraging original and innovative approaches to problem-solving, engineering design, and scientific idea generation.^11,12^ The use of food as a contextual anchor has become increasingly prevalent in STEM education in recent years, especially in high school and introductory undergraduate curricula.^13–15^

In this curriculum, we take chocolate, a popular and familiar commodity with easily accessible properties, and relatively low cost, as the model material to teach the MSE framework of “structure, process, properties, and performance” relationships from the atomic to macroscopic scale. Notably, the fascinating polymorphic characteristics of cocoa butter and the diversity of additives in chocolate offer a rich platform for in-depth exploration, allowing for many paths for student-led final projects as well as potential areas of growth to develop the curriculum into a stand-alone entry-level college curriculum.

Commercial chocolate typically consists of four main components: cocoa butter, sugar, cacao nibs, and surfactants, such as lecithin. Variations in these ingredients account for a wide range of chocolate products familiar to the public. Chocolate has been used in public-friendly demonstrations to explain scientific concepts like phase transitions and emulsification.^16^ For the MSE curriculum, however, cocoa butter is the most relevant component. It displays fascinating polymorphism, forming six different crystalline structures (I–VI) that strongly influence chocolate’s snap, gloss, and mouthfeel. Among these, form V is the most desirable, as it provides the ideal properties and is the primary goal of the tempering process.^17^ Investigations exploring the polymorphism of chocolate offer intriguing and accessible pathways to engage students with primary literature in the field.^18,19^

Our learning goals for this short course are 3-fold:

1. To create a low-barrier pathway to introduce students to the field of MSE and the research practice of asking questions, testing hypotheses, and analyzing data.
2. To foster STEM literacy and improve students’ self-perception in STEM disciplines.
3. To introduce basic coding skills and demonstrate the broad utility of the skills.

The program emphasized parallels between classroom or low-budget explorations and the more advanced experimental techniques used in research laboratories (e.g., differential scanning calorimetry, polarized optical microscopy). Students were introduced not only to the experimental mindset of MSE but also to computational tools—including coding in Python for quantitative analysis, hypothesis testing, and data visualization—that extend beyond the immediate subject matter. Coding in Python has previously been incorporated into curricula to support learning in various STEM fields, and here we extend this approach further into the area of materials science.^20–22^ By combining accessible experiments with computational approaches, the program encouraged students to see themselves as “citizen scientists,” capable of connecting everyday experiences with the practices of professional researchers. Ultimately, we aim to inspire greater interest in MSE among students at the high school and entry-college levels, while equipping them with broadly transferable analytical skills.

### Curriculum Overview

This curriculum was implemented in the Princeton University Materials Academy, a 3-week summer program on Princeton University’s campus attended by 17 high school students entering grades 10–12. During the 3 weeks, the students had opportunities to interact with research professors, research staff, and graduate students through lectures, research talks, demonstrations, and lab tours. The interactive lectures and lab activities introduced the essential concepts, such as chocolate composition, crystal structure, phase changes, microscopy, and solid mechanics, with a capstone student-led group project. The curriculum anchors the lecture, lab, and coding activities to the MSE central paradigm of “structure, process, properties, performance” interrelationships, starting with the molecular building blocks in chocolate (such as triglyceride molecules) that influence their crystallization and assembly into structures at larger scales. The curriculum examined the critical tempering process of chocolate that impacts the nano- and microstructures formed and, hence, the thermal and mechanical properties of chocolate, which, in turn, influences how the chocolate performs. This approach, summarized in Table 1, also highlights the underlying theme of scales, from molecular building blocks to mesoscale assemblies to the macroscopic product that we can see, touch, and feel (Figure 1).

**Table 1:** Overview of Course Lecture, Lab, and Coding Activities

MSE Theme	Lecture	Hands-on Activity	Coding Activity	Theme
Structure	Crystalline structures; Polymorphism in chocolate; Molecular conformation	Understanding and building triglyceride molecule; Configurations and assembly of triglycerides	Introducing ″variables″ in chocolate composition	Single molecules
Process	Conching chocolate; Tempering process	Cocoa butter crystals under microscope; Chocolate tempering	Introducing ″functions″ in fermentation and roasting	Bulk assembly
Properties	Intro to solid mechanics; Intro to microscopy; Intro to phase changes	Thermal properties: Melting Point; DSC measurements; Mechanical properties: Fracturing; Indentation	Plotting and data visualization of properties	Macroscopic properties
Applications	Chocolate composition	Desirables: taste, appearance, feel, health impact, sustainability, etc.		Performance

**Figure 1:**
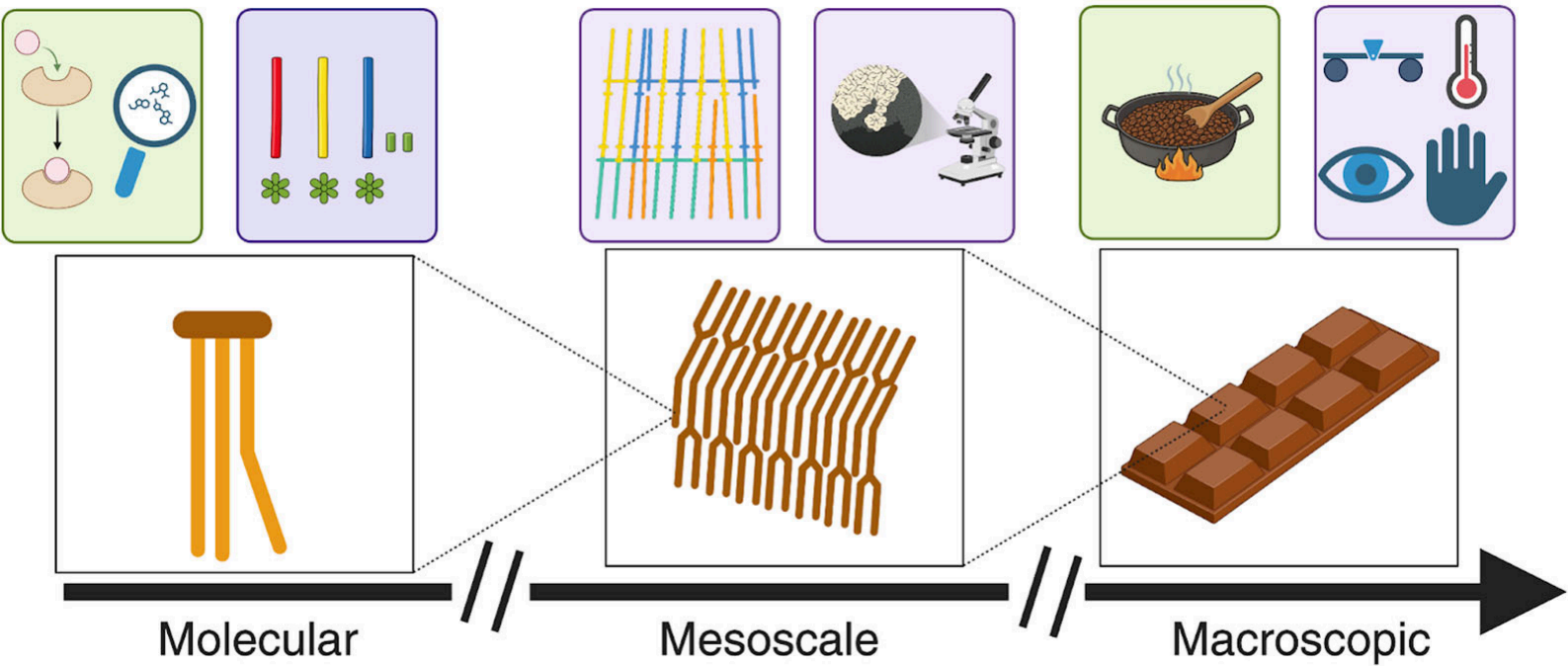
Introduction to MSE across length scales. Schematic overview of the course content across length scales ranging from molecular to macroscopic, combining experimental (purple box) and computational (green box) activities. Image was created using BioRender.

## Course Content

Here, we describe the hands-on and coding activities that were formative to the students’ understanding of core concepts and in the design of the student-led projects. A detailed description of all of the program activities is found in the .

### Introduction to Sensory Tests and Terminology

The program started with an observation-based activity, where students examined various commercial chocolate samples, including as-purchased and untempered milk chocolates. As the students broke, tasted, and handled the samples, they shared observations about taste, texture, glossiness, color, hardness, stickiness, and fracture behavior. The comparison between the tempered and untempered chocolate highlighted that even a material with the same composition can exhibit drastically different properties due to how it was processed: from shiny and brittle to matte, soft, and melty. Then, a one h workshop led by a chocolate maker introduced students to some of the historical and cultural significance of cacao and chocolate and demonstrated key processing steps and terminology such as conching, tempering, and molding. Through these introductory activities, students explored chocolate’s ingredients, desirable qualities for consumers and manufacturers, and how processing influences cocoa butter’s crystalline structures and, ultimately, the chocolate’s properties and performance.

### Molecular Modeling and Packing

To build intuition about how structure and processing affect macroscopic properties and performance, we began the curriculum at the molecular level with a low-cost model-building activity to closely examine cocoa butter (triglycerides). Triglycerides, also known as triacylglycerols (TAGs), are composed of a glycerol molecule and three fatty acids linked by ester bonds (Figure 2a). Using straws and toy connectors, students constructed triglyceride molecules and assembled them into larger structures to explore the packing. The chemical structure of a TAG molecule is simplified to four connected sections of straw of varying lengths and different colors. Students then explored the different conformations of the TAG molecular model by rotating the connectors to form the “E”, tuning fork, chair, and propeller conformations (Figure 2b-e). Then, the bilayer (Figure 2f) or trilayer (Figure 2g) chain assemblies that TAG molecules typically assumed were built with their TAG straw models (Figure 2h). Finally, stacks of sheets were put together to represent the formation of the lamella (Figure 2i-j).

**Figure 2:**
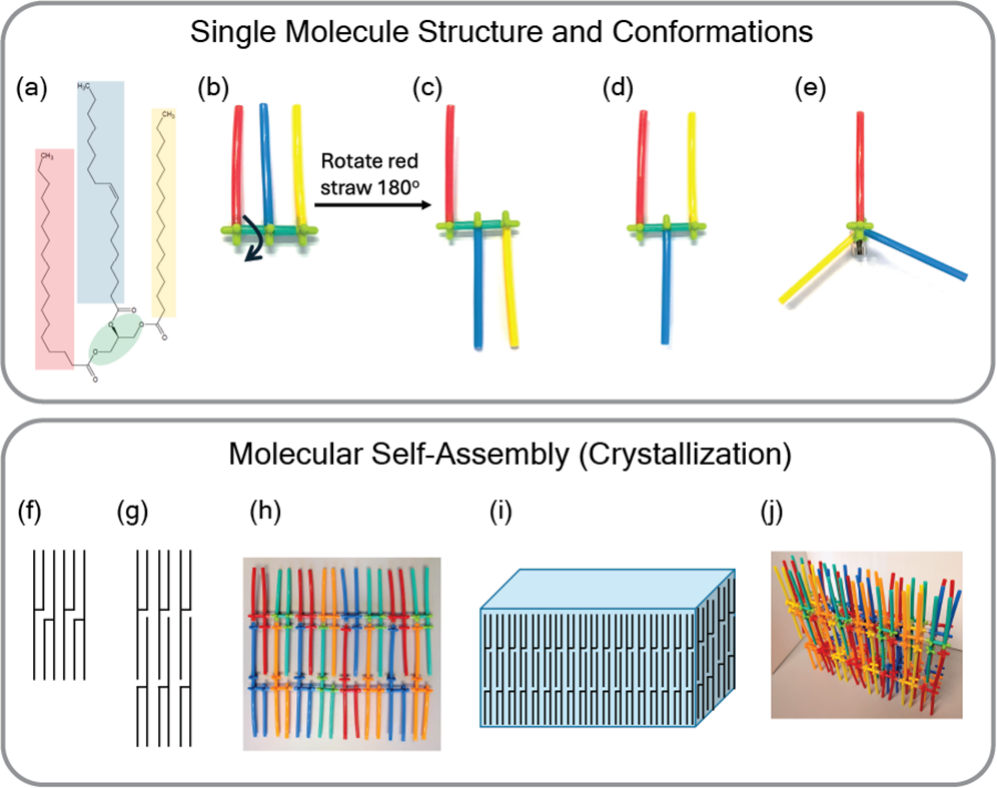
Using a simple straw model to teach triacylglycerol (TAG) molecular structure, molecular conformations, and molecular self-assembly. (a) Structure of 1-palmitoyl-2-oleoyl-3-stearyl-*sn*-glycerol (POS). (b-e) Straw model representations of the different TAG conformations: (b) “E”, (c) chair, (d) tuning fork, and (e) propeller. A change in conformation can be achieved by rotating a straw about its connector as shown in (b-c). Illustrations of TAG molecules in the chair conformation, showing (f) bilayer and (g) trilayer assembly of TAG chains. (h) Straw model representation of a single trilayer assembly of TAG chains. (i) Illustration of a lamella of trilayer TAG molecules. (j) Straw model representation of a TAG lamella.

Though simplified, these exercises introduced the chemical structure of the triglyceride molecule and allowed students to recognize that a single triglyceride molecule can adopt different conformations. Moreover, though this simple straw model cannot capture the complex subcell crystal lattice structures found in real cocoa butter, it illustrates the hierarchical nature of assembly in chocolate, from the intramolecular arrangement of chains in individual TAG molecules, to the intermolecular arrangement of TAG molecules into bilayers or trilayers of chains, to the supra-molecular assembly of lamella. In class discussions, the students learned that TAG lamellae continue to assemble into platelets, which can be observed with advanced imaging techniques, such as transmission electron microscopy (TEM), and platelets assemble into spherulites, which can be observed using optical microscopy techniques. The random network of spherulites comprises the chocolate matrix and governs the properties of the chocolate.

### Microscopic Crystal Growth

With some understanding of the molecular and supra-molecular structures in cocoa butter, we explored the larger chocolate matrix that can be directly visualized through polarized optical microscopy (POM). POM offered a great opportunity to introduce concepts such as polarized light, crossed polarizers, the difference between isotropic and anisotropic materials, and birefringence. Students built their own low-cost polarized light imaging setup from a homemade budget-friendly kit (, see Supporting Information for details) and used the setup to observe the crystallization of cocoa butter between crossed polarizers. They observed that the molten sample initially appeared black, but over time, bright white spots emerged and expanded, signaling the formation and growth of spherulites large enough to be seen with a magnifying glass or a USB digital microscope (Figure 3b-d). Observations over several days indicated that the spherulites continued to grow, exhibiting fascinating patterns and colors. Complementary demonstrations with a research-grade polarized optical microscope allowed the students to observe cocoa butter samples with the finer features of the spherulites.

**Figure 3:**
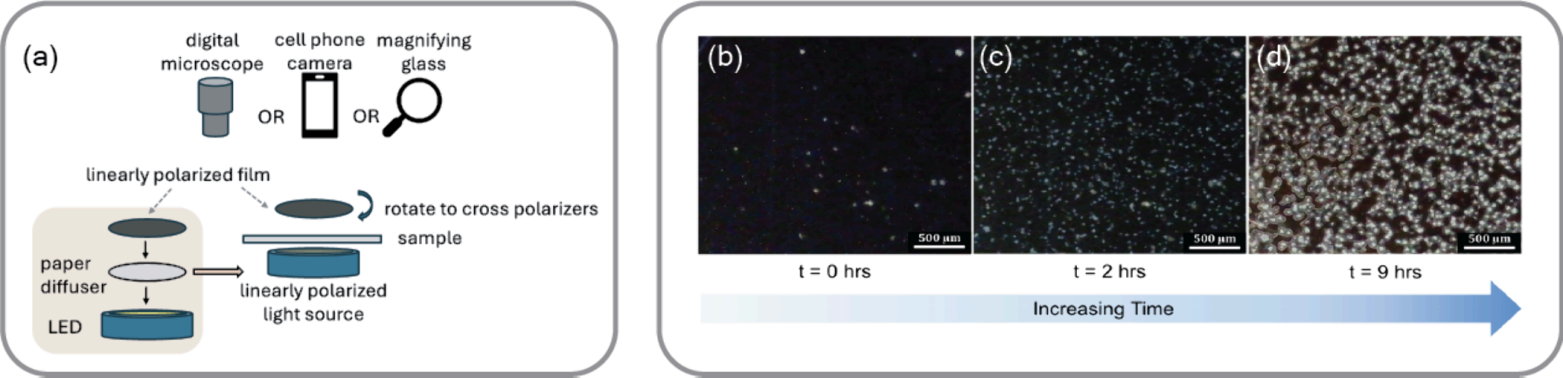
Microscopic imaging of cocoa butter with crossed polarizers. (a) Making a polarized light source for observing samples between crossed polarizers. Time series of a molten cocoa butter sample during solidification, where (b) time = 0 h, (c) time = 2 h, and (d) time = 9 h.

### Macroscopic Property Tests

To make the connection between the nano- and microscopic structures of chocolate, the macroscopic properties, and the process, we carried out two types of characterization. First, the students investigated the melting behavior of tempered and untempered chocolate using a double-boiler, while the temperature of a stirred chocolate sample was recorded periodically. Students plotted the temperature as a function of time for both samples and examined the differences in the profiles (Figure 4a). The students observed a clear plateau (or significant decrease in slope) near 34 °C in the tempered sample, indicative of the melting point, in agreement with that of Form V of the cocoa butter. In contrast, the untempered chocolate melted immediately and could not determine a melting point, suggesting a mixture of the six crystalline forms of cocoa butter. Using a differential scanning calorimeter (DSC),^23^ students carried out complementary experiments on the same samples (Figure 4b). Ten to 20 milligrams of chocolate samples were heated and cooled at a rate of 5 °C/min to confirm the melting points of the two samples, indicated by the peaks in these plots. Similarly, DSC revealed a melting point at around 33 °C in the tempered sample and a broader phase transition below 20 °C in the untempered sample. These characterization tools were later frequently applied in students’ final projects to infer which crystalline forms of cocoa butter were present, as described below in Student Outcomes.

**Figure 4:**
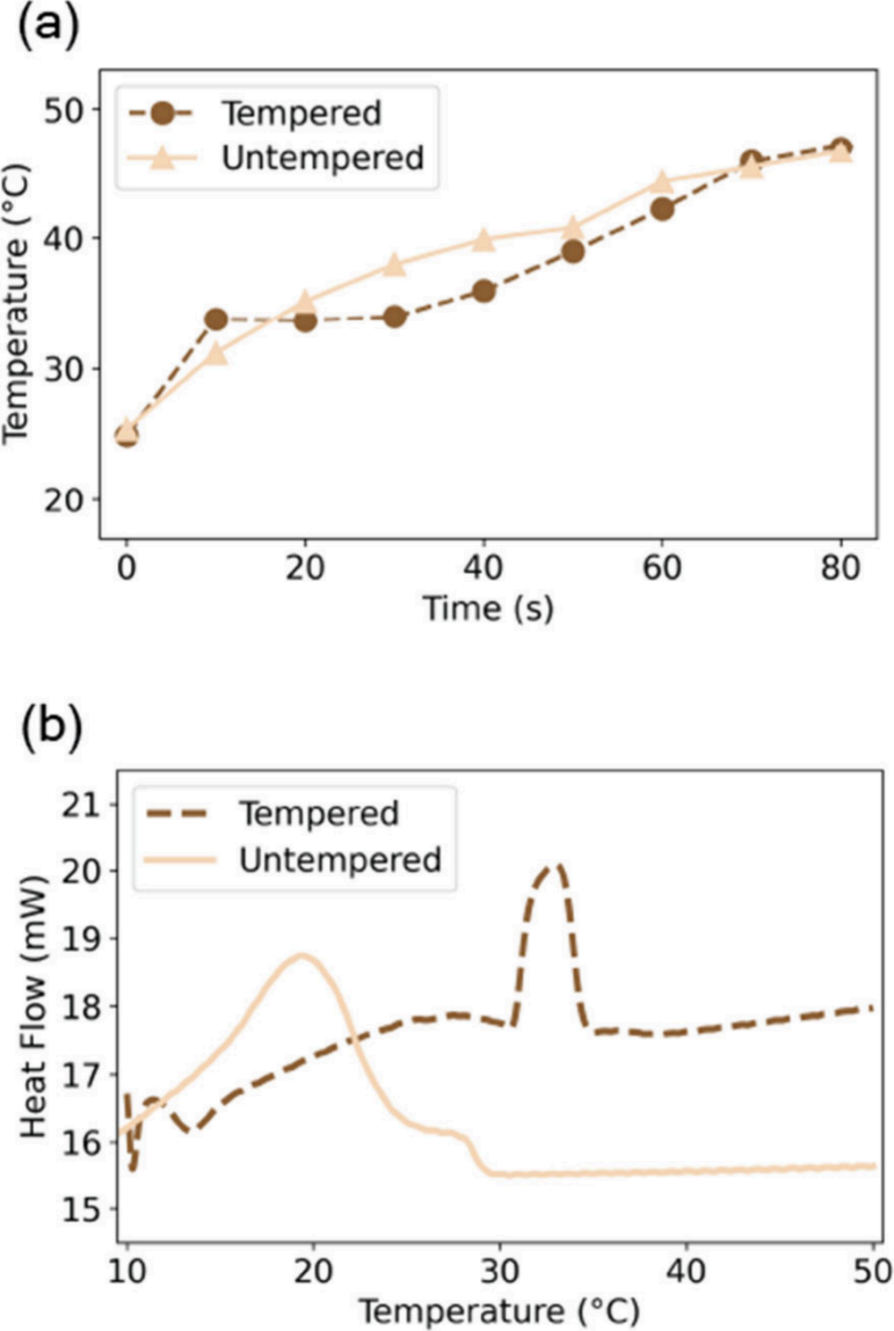
Melting characteristics of chocolate. (a) Temperature of tempered and untempered milk chocolate samples as a function of time when heated to 55 °C, showing a melting transition for tempered chocolate at around 34 °C. (b) DSC thermograms of the same tempered and untempered milk chocolate samples as in (a) showing melting peaks around 33 and 19 °C, respectively.

Mechanical property characterizations of chocolate have been well-publicized with several innovative approaches.^24–28^ In our curriculum, students explored 3-point bending tests and indentation tests on tempered chocolate, executing procedures that can be done at home and in the classroom (refer to for details). By hanging weights from a chocolate bar suspended between two supports until it broke, as illustrated in Figure 5a, the students estimated the flexural strength of a chocolate bar.^28,29^ The indentation test characterized the hardness of chocolate by measuring the size of the indentation formed when a falling metal sphere impacted the chocolate, dropped from known heights.^30^ Students combined their data to investigate the correlation of the indentation diameter and the falling height (Figure 5b). As the falling height increases, the indentation diameter also increases. The lab activities provided quantitative approaches to investigate the “snap” and deformability of well-tempered chocolate. Students implemented these types of characterizations on the chocolate samples in their final projects, as seen in Student Outcomes.

**Figure 5:**
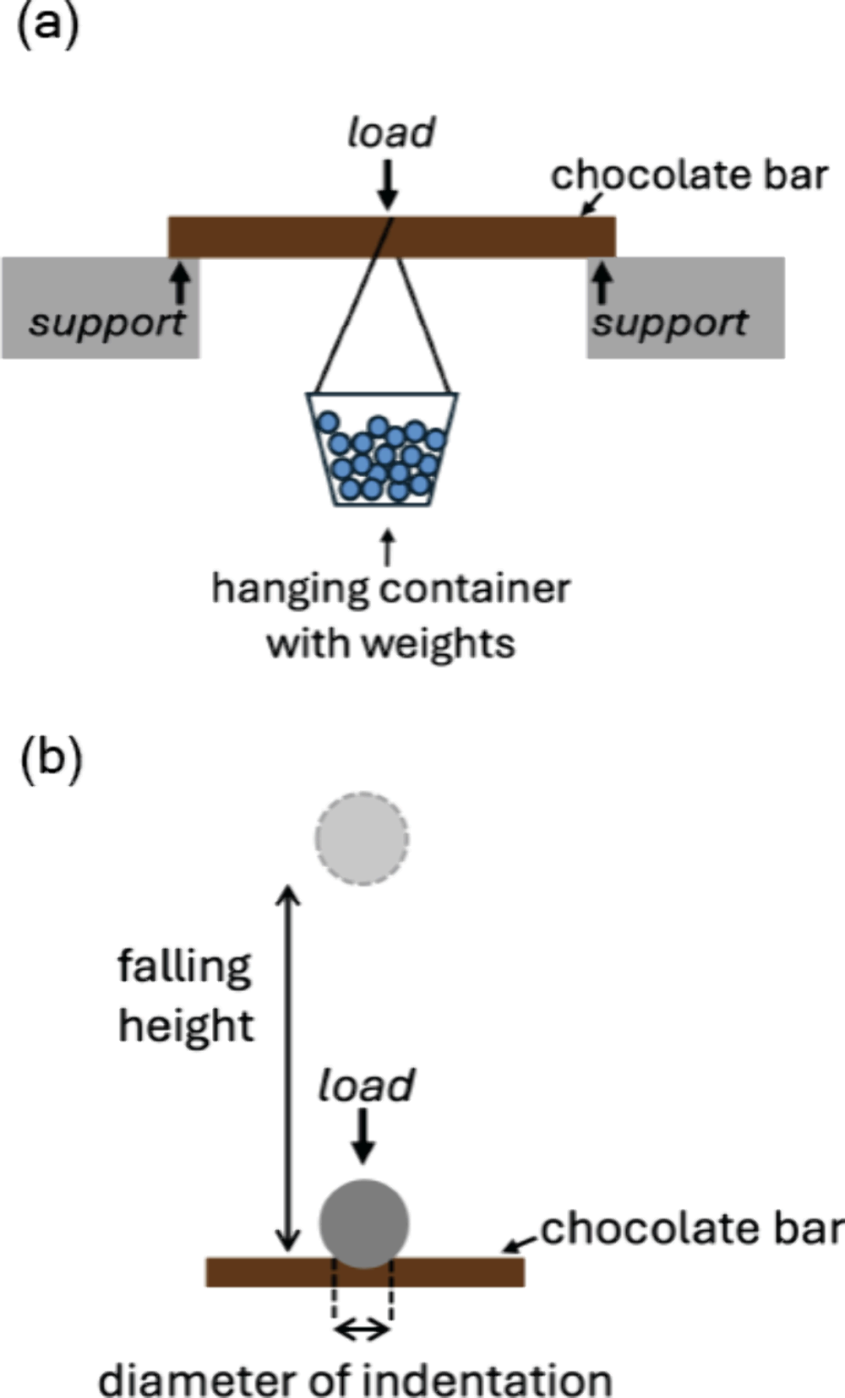
Characterization of the mechanical properties of chocolate. (a) Illustration of the three-point bending test where a load (a hanging container filled with weights) is applied to the center of a chocolate bar supported on both ends. (b) Illustration of the indentation test, where a stainless steel ball (diameter = 28.3 mm) is dropped from various heights and impacts a chocolate bar, leaving an indent.

### Python Coding Incorporation

Although students often associate learning Python coding with a career in Computer Sciences, we want to demonstrate the wide application of this skill in MSE research and improve their competency and perception in coding. The complete Notebook of the Python exercises is shared in the .

We began by introducing the concept of variables framed through the chemistry of chocolate. Students learned that a variable in Python functions like a labeled container that stores information—mirroring how chemists record and track quantities of ingredients. Using chocolate as a context, students created variables to represent concentrations of key bioactive molecules (e.g., theobromine, serotonin, tryptophan, and phenylethylamine) in different units. With the defined variables, students then practiced basic operations (addition, subtraction, multiplication, division) to simulate how these compounds might contribute to chocolate’s physiological or mood-enhancing effects. They also explored data types (integers, floats, strings, and Booleans) and identified the type associated with each variable. To demonstrate mastery, students completed small challenges: estimating a “chemical load score” by combining variables, storing descriptive text about chocolate’s effects on brain chemistry, and designing a new “compound” with its own variable name, concentration, and effect.

We next introduced Python functions by framing them as reusable procedures for modeling stages of cocoa pulp–bean fermentation. Building on prior work with variables and the four basic operations, students defined small, single-purpose functions that mirrored lab ideas: combining sugar sources (pulp + bean), tracking enzyme activity and loss during heat exposure, estimating simple energy terms from microbial counts, and normalizing activity per gram of cocoa mass. The focus on enzymes was added at the student’s request, leveraging interest to deepen engagement with chemistry.

Pedagogically, we used a “board-first, code-second” approach: students mapped inputs, outputs, and units and then translated those relationships into functions with parameters and return values. Practice tasks (e.g., enzyme_total, enzyme_loss, fermentation_energy, enzyme_per_gram) emphasized parametrization, arithmetic composition, and checking results with test values. A capstone prompt asked students to build a process_score that sequentially combined addition, subtraction, multiplication, and division to integrate sugars used, enzyme factors, and fermentation time—an exercise in decomposition, abstraction, and composition without introducing new syntax beyond functions. These exercises positioned functions as tools for scientific modeling: students reasoned about units and plausible ranges, articulated assumptions, and saw how small, validated functions can be chained to model a complex materials-processing workflow.

In the final module, students advanced from variables and functions to using Python visualization libraries (e.g., matplotlib, seaborn) to generate professional-quality plots.^31,32^ Framed within chocolate processing, they used synthetic data to plot flavor score versus fermentation duration, flavor intensity by roasting temperature (Figure 6a), aroma intensity versus particle size (Figure 6b), distribution of conching times, and cocoa butter crystal stability. The examples included line graphs, bar charts, scatter plots, histograms, and box plots, giving students practice with diverse forms of data representation.

**Figure 6:**
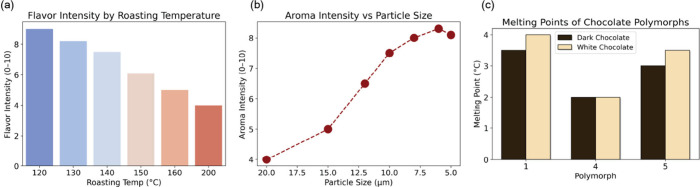
Visualization of the chocolate material properties. In the graphing and visualization Python module, students learned how to create various types of plots through the context of chocolate processing. For example, they used synthetic data to learn how to plot (a) a bar graph for flavor intensity by roasting temperature and (b) a scatterplot for aroma intensity versus particle size. (c) They also used synthetic data to create templates of other types of graphs, such as a grouped bar chart, which they could later modify for their final projects.

Students were encouraged to experiment with coefficients, add conditions (e.g., oxygen-rich vs oxygen-poor fermentation), and vary parameters to see how outcomes shifted visually. We emphasized producing publication-quality figures by adjusting parameters such as resolution (dpi = 300), color palettes, axis labels, and inverted axes where appropriate. This iterative approach helped them develop a graphing “template library” (see Figure 6c for example) using synthetic data they could later adapt for analyzing their final project. For their final projects, students applied these skills to construct original visualizations, reinforcing both chemical understanding and computational literacy. These exercises highlighted how visualization can function as both a tool for analysis and a medium for scientific communication, giving students a glimpse of the broad utility of coding in MSE research

## Student Outcomes

We evaluate the effectiveness of the short course in light of our Learning G oals in two ways: (1) student-led final projects and (2) pre- and poststudent surveys.

### Student-Led Final Projects

Students in groups of two were assigned to design and execute a final project, a major deliverable of the curriculum. They were tasked to have a clearly stated hypothesis, identify variables and characterization methods, and visualize their findings by using Python programming. Although not graded in the traditional sense, the student final projects served as a tool to gauge their familiarity with the MSE research process and methods. The projects were evaluated in two folds: (1) students’ ability to formulate and execute a project following the MSE research process and (2) their use of Python to visualize experimental data. These criteria align with Learning Goals 1 and 3 outlined above.

The final projects were developed in 4 stages:

1. Propose a research question and identify at least one primary literature relevant to their research question.
2. Form a hypothesis based on their reading and develop a procedure with a supply list.
3. Peer-review the proposed procedure and revise based on feedback.
4. Execute the project under supervision.

Based on the submitted procedure and supply list, students were provided with the necessary materials and equipment to execute their projects. A detailed description of the supplies and equipment for the final projects is provided in the . The project findings were shared in a public poster session and in written reports. Table 2 summarizes these projects.

**Table 2:** Summary of Student-Led Projects

Variables	Property Studied	Python Visualization
Types of fat (e.g., palm oil, shea butter)	Flexural strength, Melting point, Microscopic observations, Sensory test	Scatter plot
Types of crystal seed during tempering	Flexural strength, Malleability	Grouped bar plots, Scatter plot
% salt and other additives	Conductivity	Grouped bar plots
Polymorph crystalline structures	Flexural strength, Melting point, Sensory test	Grouped bar plots
Inclusion of liquids (e.g., corn syrup)	Flexural strength, Melting point	Grouped bar plots, Scatter plot
Inclusion of additional oils (e.g., coconut oil)	Flexural strength, Melting point	Grouped bar plots
Inclusion of cacao nibs (white vs dark chocolate)	Flexural strength, Melting point, Sensory test	Grouped bar plots
Inclusion of solids (e.g., almond)	Flexural strength	Grouped bar plots

Through these final projects, students demonstrated understanding of the scientific process and significant growth in materials science skills and STEM literacy. They showed proficiency in generating hypotheses grounded in primary literature and tested these ideas by examining connections among structures (variables), properties, and performances, thereby applying the MSE framework. The course activities equipped them with practical experience in characterization techniques, including the 3-point bend testing, indentation, DSC, and microscopic evaluation. These characterization methods were widely applied in their final projects, providing them with adequate tools and allowing them to collect authentic data to investigate their hypotheses using materials science approaches. For instance, students observed spherulite formation from cocoa butter, coconut oil, and palm oil to evaluate the impact of different fats in the ingredients using polarized optical microscopy. Students conducted the 3-point bending test to assess the impact of inclusions of liquid and solid ingredients on the flexural strength of the chocolate bars. Students also attempted measuring melting points of chocolates by using DSC when different cocoa butter crystalline forms were present by controlling the tempering process. Lastly, students applied their newly acquired coding skills through Python training to analyze and visualize the data for their final projects.

### Student Survey Results

A survey, based on the Student Attitudes toward STEM (S-STEM) Survey^33^ with additional questions to assess coding proficiency, was given to the students on the first day of the program and readministered on the last day before the poster presentations. Seventeen students completed both the pre- and post- surveys (n = 17). From the survey, we selected questions that best aligned with our teaching objectives and compared student responses before and after the program.

Pre and post increases were observed across measures of coding experience, coding confidence, and perceptions of coding in science, as well as in broader constructs including engineering perception, math and science utility, engineering utility, and math potential (Figure 7). While changes in coding experience were statistically significant (*p* < 0.05) and coding confidence approached statistical significance (*p* < 0.1), other measures did not reach significance (*p* > 0.1). Since survey items were measured on a 5-point Likert scale, responses were treated as ordinal data, and changes were evaluated using nonparametric paired Wilcoxon signed-rank tests. Effect sizes, calculated as rank-biserial correlations (r), were medium to large (r = 0.2–0.45 for STEM literacy and self-perception, r = 0.88–0.94 for coding), suggesting meaningful educational impact despite the limited sample size.

**Figure 7:**
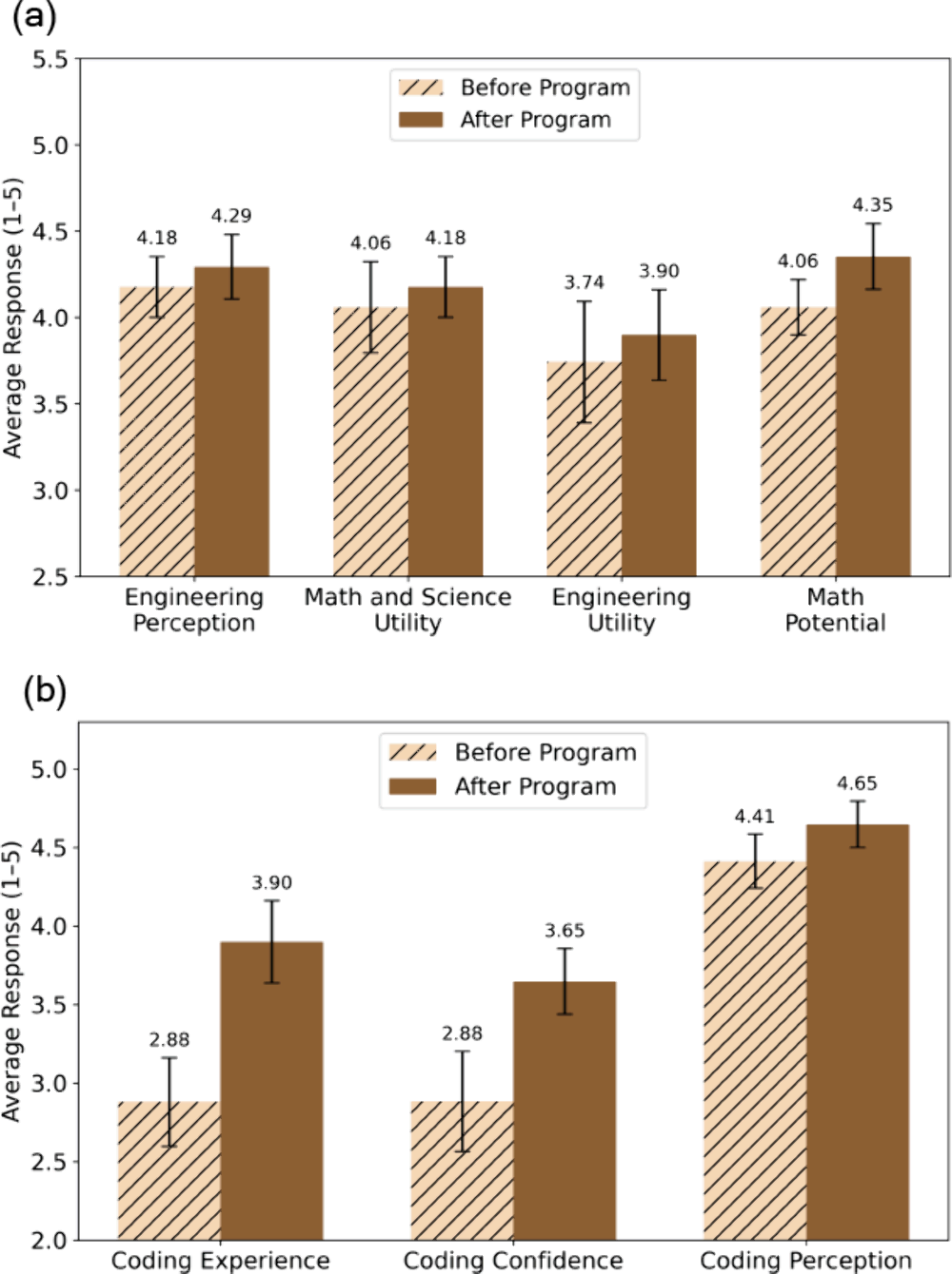
Results from student survey responses show improvement in (a) STEM literacy and self-perception and (b) coding after the program. All survey questions were ranked on a Likert scale and converted to numerical values ranging from 1 to 5 for quantitative comparison and plotting purposes. Bars represent mean Likert responses with ± standard error. The mean values for before and after the program are labeled above each bar. Statistical significance was assessed using a Wilcoxon signed-rank test (*n* = 17). The complete questions and conversion can be found in the Supporting Information.

Cronbach’s alpha values, calculated to measure internal consistency of the results, were α = 0.763 (before) and α = 0.748 (after) for STEM literacy and perception and α = 0.663 (before) and α = 0.804 (after) for coding. For STEM literacy and perception, the reliability falls into an acceptable range. For coding, the reliability before is moderate but acceptable, and the reliability after is good, showing that the student responses were more consistent after the program, which can occur if they better understand the constructs being measured. Figure 7(a) summarizes answers to items probing students’ beliefs about the usefulness of STEM knowledge, their confidence in applying it, and their self-perception of future ability, corresponding to our second Learning Goal of fostering students’ self-perception in STEM disciplines. Postprogram responses were consistently more favorable, with the most notable shift seen in students’ confidence in tackling more advanced math problems — even though none of the lectures or activities explicitly emphasized mathematical skills. Similarly, survey questions related to the coding component (Figure 7(b)) revealed substantial growth in students’ competency, confidence, and perception of Python coding. While only 37% of the class had prior coding experience, by the end of the program, all of the students demonstrated enough proficiency to visualize data using Python plots in their final projects. The overall improvements in students’ attitudes toward STEM, their awareness of engineering practices, and their confidence in developing skills for the future are very encouraging.

Taken together, both the student-led final projects and the survey analysis indicate our Learning Goals in building a pathway to the MSE discipline and research approaches, improving students’ self-perception in STEM, and introducing coding skills have all been successfully achieved through this short course.

## Future Directions and Conclusions

By grounding abstract materials science concepts in a familiar context of chocolate, this short course curriculum achieves our learning goals of creating a low-barrier pathway to the field of MSE and strengthening students’ scientific literacy. The program’s modular design also makes the curriculum easily adapted into existing courses or expanded as a new stand-alone course.

Specifically, at the molecular level, the simple straw models of TAG could be enhanced by incorporating unsaturated bonds and kinks into the fatty acid chains by using additional connectors. This modification would allow students to observe how fatty acid diversity influences molecular packing and, in turn, crystallinity. Additional computational chemistry modeling of the TAG molecules (using Python packages such as RDKit and BuildAMol) could also aid 3D visualization of the molecules and further reinforce concepts of packing and organization. In addition, students can be introduced to diffraction-based techniques for crystalline material characterizations through the primary literature and lectures with deeper exploration. This module can be enhanced by incorporating activities from the educational literature^34^ to strengthen connections to introductory physics and to provide a more thorough understanding of diffraction. Lastly, concepts in rheology^35–37^ could likewise be integrated, particularly in the context of tempering and the addition of emulsifiers, to emphasize viscosity as a key material property.

This curriculum reflects our commitment to demystifying research: making the methods and reasoning of materials science transparent to nonspecialists, so that the field feels less hidden and more approachable. Activities were designed to draw parallels between everyday experiences and underlying material phenomena, effectively “opening the black box” to introduce students to authentic MSE research practices. By explicitly connecting simple activities to advanced experimental methods, students were able to appreciate both the utility of instrumentation and the continuity between at-home explorations and laboratory science. The approach highlighted that while professional research tools provide higher resolution and more information, the core principles are accessible and not fundamentally elusive.

By working across multiple length scales—from the molecular composition of cocoa to mesoscale transformations during processing and macroscopic properties like texture and stability—students developed an integrated perspective on process–structure–properties–performance relationships. In this way, the curriculum not only introduced students to computational and experimental practices but also modeled how materials scientists investigate the material world around us.

## Supplementary Material







## Notes

Student Handouts, Survey Instrument, Instructor’s notes, and Program Schedule (PDF)
Student Handouts, Survey Instrument, Instructor’s notes, and Program Schedule (DOCX)
Jupyter notebook for Python modules (ipynb) (ZIP)
