# Supplementary material for: The Chocolate Curriculum: A Gateway to Materials Science and Engineering and Python Programming
Source: J Chem Educ. 2026 Apr 15;103(5):2776–84. doi: 10.1021/acs.jchemed.5c01442 (PMC13173523; doi:10.1021/acs.jchemed.5c01442)
Supplement: Supplementary file 2 [file ed5c01442_si_002.docx]

**Supporting Information**

The Chocolate Curriculum: A Gateway to Materials Science and Engineering and Python Coding

Janine K. Nunes * ^1,2,3^ Ananya Chakravarti ^3^, Z. Vivian Feng * ^4^

1. Princeton Materials Institute, Princeton University, Princeton, NJ 08544

2. Mechanical and Aerospace Engineering, Princeton University, Princeton, NJ 08544

3. Chemical and Biological Engineering, Princeton University, Princeton, NJ 08544

4. Center on Science and Technology, Princeton University, Princeton, NJ 08544

* corresponding authors

Table of Contents

[Detailed description of the PUMA schedule 2](#_Toc220037912)

[I. Introductory chocolate observations 3](#_Toc220037913)

[II. Building triglyceride models 4](#_Toc220037914)

[III. Melting behavior of chocolate 6](#_Toc220037915)

[IV. Mechanical testing of chocolate 6](#_Toc220037916)

[V. Building a crossed polarizer light setup and imaging cocoa butter 6](#_Toc220037917)

[Final projects 9](#_Toc220037918)

[Description of Python notebook resources 9](#_Toc220037919)

[Survey Instrument 10](#_Toc220037920)

[References 11](#_Toc220037921)

[Appendix 1. Handout for Building Models of Triglycerides 12](#_Toc220037922)

[Appendix 2. Handout for Investigating the melting behavior of chocolate 15](#_Toc220037923)

[Appendix 3. Handout for Mechanical properties of chocolate 17](#_Toc220037924)

[Appendix 4. Handout for the Polarized light kit activity 23](#_Toc220037925)

## Detailed description of the PUMA schedule

This curriculum was developed for the Princeton University Materials Academy, a 3-week summer program for high school students entering grades 10-12. Students were recruited through partnerships with schools and community organizations that run academic enrichment programs for local New Jersey high school students. The program included several elements described below, which were developed to introduce high school students to materials science and engineering, as well as research. During the three weeks, high school students had opportunities to interact with research professors, research staff, and graduate students through the lectures, research talks, demonstrations, and lab tours. The program also included other social and pre-college enrichment activities (not described here).

**Lecture and classroom/lab activities:** The first week of the program focused on introductory interactive lectures, as well as classroom and lab activities to introduce the essential concepts about chocolate composition, crystal structure, phase changes, microscopy, and solid mechanics (Table S1). Since many aspects of the curriculum involved tasting chocolate samples, most activities were conducted in a standard classroom, with access to a fridge, freezer, running water, and sinks in a nearby kitchen.

**Python tutorials:** Starting on the third day of the first week, a 1-hour Python tutorial was held each day for nine days, and the final session served as an “office hour” for students seeking consultations on applying Python to data visualization in their poster presentations. For the program duration, students were provided with laptops pre-loaded with Anaconda.

**Research interactions:** The students’ experimental activities, many of which could be conducted at home, were often carried out in parallel with demonstrations using advanced research instrumentation in Princeton University’s Imaging and Analysis Center. The program included talks and interactive demonstrations with research faculty and graduate students where connections to the types of investigations that the students carried out, for example, the use of a polarized optical microscope in research on organic molecule crystallization and liquid crystals.

**Projects:** The second and third weeks of the program focused on student-led independent projects. Students worked in pairs on the design and execution of mini-projects carried out in the classroom or a teaching lab, and they could submit samples for Differential Scanning Calorimetry (PerkinElmer DSC-8500) and polarized optical microscopy (Zeiss Axio Scope A1). The *Final Project* section below (Table S3) describes the equipment and supplies available to the students for their projects.

**Poster presentation:** the students presented their projects at a poster session on the last day of the program to peers, family, friends, and university researchers.

**Table S1:** Summary of first week lab and lecture activities in the chocolate curriculum (not including the Python coding activities).

| **Day** | **Type of activity** | **Content/topic** |
| --- | --- | --- |
| 1 | Orientation | Program introduction, STEM attitudes survey, code of conduct, and lab safety |
|  | Interactive lecture | Introduction to materials science |
|  | Class activity/ ice breaker activity | Introductory observations about chocolate (section I) |
|  | Workshop | Learning about chocolate with a chocolate maker: conching, tempering, and molding chocolate |
|  | Interactive lecture | What does chocolate have to do with materials science? |
| 2 | Interactive lecture | Introduction to crystal structures |
|  | Lab | Building triglyceride models (section II) |
| 3 | Interactive lecture | Introduction to phase changes |
|  | Lab | Melting behavior of chocolate (section III) |
| 4 | Class activity | Examination of chocolate ingredients (reading labels) |
|  | Interactive lecture | Introduction to solid mechanics |
|  | Lab | Three-point bending and indentation tests of chocolate (section IV) |
| 5 | Interactive lecture | Introduction to polarized light microscopy and birefringence |
|  | Lab | Building a crossed polarizer light setup and imaging cocoa butter (section V) |
|  | Lecture and discussion | Scientific method and how to conduct a research project? |

### I. Introductory chocolate observations

In a first-day observational activity, students were given six candy bars: as-purchased milk chocolate, untempered milk chocolate, milk chocolate with almonds, milk chocolate bar with crisped rice cereal, milk chocolate bar with wafer layers, and a taffy strip candy (Figure S1). To untemper the chocolate, we left the purchased chocolate bars in the wrapper in a hot car for one day, then cooled them to room temperature; alternatively, the chocolate can be heated briefly in an oven then cooled rapidly. The students were instructed to observe and play with each candy bar, including breaking them and eating them. Then, the students shared their observations about the chocolates. The students commented on the taste, texture, glossiness, color, hardness, how the candy fractures, and stickiness. The comparison of the tempered and untempered milk chocolate highlighted that a material with the same composition can exhibit drastically different properties simply due to how it was processed: from shiny and brittle to matte, soft and melting in the hands. The various “composite” chocolate bars highlighted the mechanical differences in the chocolate bars when they were broken and chewed. The taffy candy bar was included to provide a significantly more ductile comparison to the tempered chocolate.


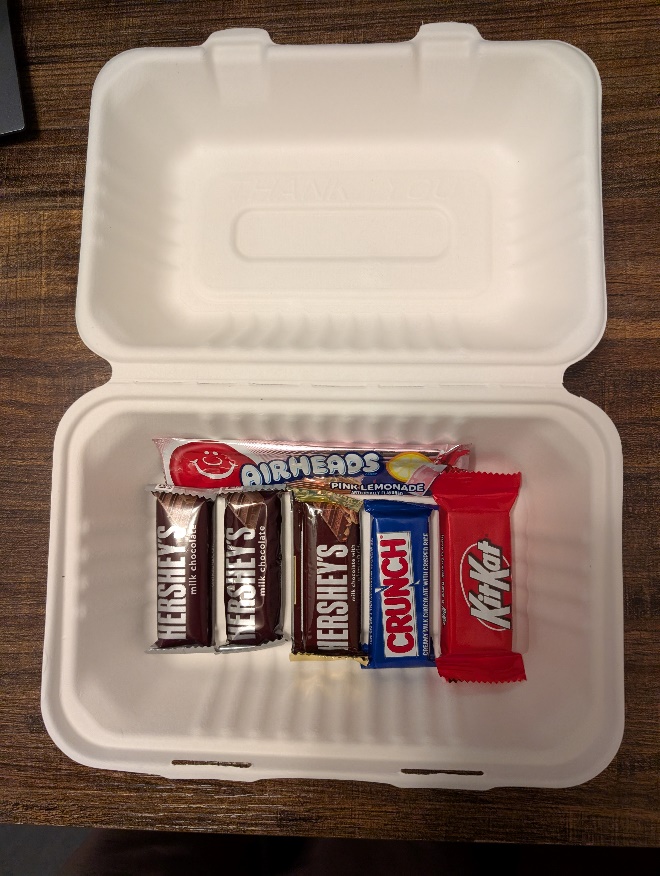


**Figure S1.** Chocolate observation kit

### II. Building triglyceride models

An inexpensive model-building activity using straws and connectors was designed to represent a triglyceride molecule and assemblies of triglyceride molecules. Lab activity kits were prepared from an inexpensive Straws and Connectors Builders Toy Set (~$28 on Amazon). Straws were cut to the appropriate length and separated with connectors into individual kits for students. Students were given a kit with eight pieces – three long straws (red, blue, and yellow) with length = 9.3 cm, two green short straws (length ≈ 1.5 cm), and three 6-pronged connectors – and asked to build a simple stick representation of a TAG molecule in the shape of an “E”. Then, students were asked to manipulate their straw TAG molecule so that it approximately represented a tuning fork, a chair, and a propeller without disconnecting any pieces of their TAG molecule. The connectors allowed for 360^o^ rotation of the three hydrocarbon straws perpendicular to the green glycerol backbone, so it was straightforward to rotate the appropriate straws to create the tuning fork and chair conformations. To create the propeller with this simplified straw system, the model must be oriented with the glycerol backbone pointing at the student. Then the three straws representing the hydrocarbon chains could be rotated to mimic the shape of a propeller.

In this simple model, all fatty acid chains are represented by straight cylindrical straws; however, a bent shape more accurately represents unsaturated fatty acid chains. This kit can be modified to incorporate this representation by including flexible drinking straws, cut to the appropriate length, as shown in Figure S2 below.


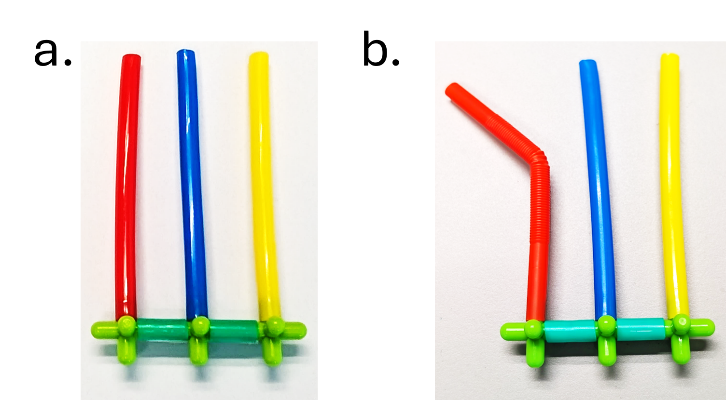


**Figure S2.** Modifying kit for exploring the conformations of triglyceride molecules. (a) Representation of TAG using straight straws. (b) Representation of TAG using a flexible straw for an unsaturated fatty acid chain.

In a second model-building exercise, students first built several TAG molecules in the chair conformation. For simplicity and sturdiness of the final assembled models, the TAG molecule representations were in a fixed chair configuration, and a single molecule was constructed using a single color. Then, working in teams of two or three, the students used the TAG building blocks to explore different ordered arrangements of the TAG molecules in a single plane. To help differentiate different TAG molecules, the students were instructed to place molecules of different colors next to each other. Following a discussion on the typical arrangement of TAG molecules into chain bilayers or chain trilayers, one arrangement was selected, e.g., trilayer, and the students worked together to assemble three or four sheets composed of at least 16 TAG building blocks, where the molecules were connected at the connectors by transparent plastic tubes into a single sheet. Short 1.5 cm long pieces of Tygon tubing (inner diameter 3/16”, outer diameter 3/8”) were used for this purpose. Multiple sheets can be stacked using clear tubes to connect them. By orienting the stack so that the chain ends point towards the student, a student could observe the pattern adopted by the chain ends. Depending on the registration of the molecules between layers, the students may observe a square, hexagonal, or random arrangement of chain ends.

### III. Melting behavior of chocolate

The students investigated the melting behavior of two chocolate samples: a tempered chocolate sample, used as purchased, and an untempered chocolate sample. Lab procedures investigating the melting of chocolate have been developed by others,^1^ and used here with modification. In brief, a double-boiler was set up with hot water (approximately 55°C). The temperature of the chocolate sample in the inner container was recorded every 10 seconds while stirring with the thermometer until the chocolate reached the same temperature as the water in the bath. The students plotted the temperature as a function of time for both samples and discussed the differences between them. Complementary demonstrations were conducted with the same tempered and untempered chocolate samples using a differential scanning calorimeter (PerkinElmer DSC-8500). 10-20 mg of each sample was placed in an aluminum pan, which was then crimped. In the DSC, the samples were heated and cooled at a rate of 5 ^o^C/min.

### IV. Mechanical testing of chocolate

Three-point bending lab experiments of chocolate using hanging weights have been previously described, and are used here with modifications.^2,3^ In brief, a chocolate bar was placed between two tables, supported under its ends. The unsupported length, width, and thickness of the chocolate were measured and recorded. A collection box was placed under the chocolate to collect the weights after fracture. Holes were punched in a plastic cup, and twine was used to hang the cup at the center of the chocolate bar. Weights (marbles, steel balls, and pennies) were added gently and gradually to the cup until the chocolate fractured. The mass of the weights, cup, and twine was measured on a balance and recorded. The flexural strength of the chocolate sample was calculated.

Indentation lab experiments of chocolate are based on previously developed STEM outreach activities.^4^ In brief, a chocolate bar (smooth side) was placed on a flat surface. A stainless steel ball with a diameter of 28.3 mm was dropped from a known height guided by a rolled paper tube. The diameter of the indentation was measured and recorded. Students investigated the size of the indentation as a function of height.

### V. Building a crossed polarizer light setup and imaging cocoa butter

Kits were prepared for the students to build their own low-cost polarized light imaging setup. The kits included a small, inexpensive LED light source with a flat top surface, a paper diffuser if the light source was not already diffuse, adhesive-backed linear polarizing film, non-adhesive polarized film (or polarized glasses) all cut to the approximate size of the LED surface, and a magnifying glass or an inexpensive USB digital microscope. A parts list is provided in Table S2. The student adhered the diffuser (if needed) and then applied the adhesive polarizing film to the surface of the LED. Using the second non-adhesive film or polarized glasses, the students observed various transparent or translucent objects between crossed polarizers to identify which were birefringent and could be observed with their setup. For example, they observed polyethylene film (from a plastic bag) before and after stretching (stress-induced birefringence), and compared it to a bare microscope slide, which is not birefringent, and to microscope slides already prepared with anisotropic crystal films.

To visualize cocoa butter crystallization at the microscale, the students prepared cocoa butter microscope slide samples by heating cocoa butter to 50°C for 10 minutes, then sandwiching a small drop of the molten cocoa butter between a standard microscope glass slide and a cover glass. The students observed the molten sample between crossed polarizers as it solidified at room temperature with magnifying glasses. Images were also captured with a 3840 x 2160 pixel digital microscope camera.

**Table S2.** Parts list for polarizing light set up kit

| **Part** | **Example** | **Approximate cost (per kit)** | **Notes** |
| --- | --- | --- | --- |
| Light | EZVALO LED rechargeable puck lights, available on amazon.com | $3.90 | Different lights were evaluated for this kit. A good, though lower light intensity, alternative is the Adafruit 45 mm x 86 mm backlight module. It is already diffuse, however separate power (e.g., a battery pack) must be purchased. |
| Adhesive linear polarized film | Selens Polarizing Film 7.8 x 11.8 inches Adhesive Polarized Sheets | $0.50 | Cut into 4.5 cm squares |
| Non-adhesive linear polarized film | Selens Linear Polarizing Film A4 7.8x11.8 inch Non-Adhesive Polarized Film Sheets for Physics Education | $0.50 |  |
| Diffuser | White shipping labels cut to size | $0.03 | 1. Non-adhesive printer paper works well, and can be adhered to light with tape.  2. If using Avery shipping labels, do not use those with TrueBlock Technology®; too much light is blocked |
| Polarizing paper glasses | Rainbow Symphony 3D Polarized Glasses Linear Polarization Paper Frames | $0.50 | Can be used in addition to or as an alternative to the non-adhesive linear polarized film |
| Magnifying glass |  | $2-$4 | If plastic magnifying glasses are used, the glasses may be birefringent and visible when used with the polarizing glasses |

## Final projects

To execute their projects, students had access to all the equipment and supplies provided for the class and lab activities described above, including various commercial chocolate bars.

**Table S3.** Equipment, materials and supplies available to students for final projects

| **Equipment** | **Notes** |
| --- | --- |
| Chocolate warmers with adjustable temperature control in the range 30 - 85 | $60 - $85 each, depending on model. There were two warmers available for the students. Each warmer contained two tanks or pots, so four different samples could be prepared simultaneously. |
| Two handheld electric mixers | $10 or more each, depending on model. Useful for mixing ingredients, e.g., when preparing chocolate from cocoa powder, fat, sugar, and milk powder. |
| Silicone chocolate molds | (~$1.40 each) Molds were 6.3 x 3.1 x 0.28 inches, with a 6x4 break-apart rectangular grid. The mold capacity was ~3 oz. |
| Balances | One per student group |
| Other miscellaneous kitchen utensils | Spatulas, spoons, bowls, trays |
| Cocoa butter and chocolate for tempering | - Cocoa butter - Chocolate bars: dark (100%, 90%, 85%, 70%), milk and white chocolate - Chocolate baking wafers with high cocoa butter content (bittersweet, semi-sweet, and milk) |
| Other ingredients (general stock and student requests) | Cocoa powder, powdered sugar, milk powder, various fats (e.g., shea butter, palm kernel oil, coconut oil, hazelnut oil), corn syrup, almonds, carrots, shredded coconut, salt |

## Description of Python notebook resources

The Python notebooks which we built are available as Jupyter notebook files (Module_1.ipynb, Module_2.ipynb, Module_3.ipynb). Module 1 is on variables with the topic of chemical composition of chocolate, Module 2 is on functions with the topic of chocolate fermentation and enzymatic reactions, and Module 3 is on plotting and visualization with the topic of chocolate processing.

## Survey Instrument

The survey question prompts and answer choices that are mapped onto a Likert scale for analysis in Figure 7 of the Main Text are as follows. Conversion to numerical values from 1 to 5 is indicated in parentheses next to each answer choice.

The first four questions come from the S-STEM survey.^5^

**Engineering Perception:**

Question prompt: If I learn engineering, then I can improve things that people use every day.

Answer choices: Strongly Disagree (1), Disagree (2), Neither Agree nor Disagree (3), Agree (4), Strongly Agree (5)

**Math and Science Utility:**

Knowing how to use math and science together will help me to invent useful things.

Answer choices: Strongly Disagree (1), Disagree (2), Neither Agree nor Disagree (3), Agree (4), Strongly Agree (5)

**Engineering Utility:**

Question prompt: People use science, math and computers to build different products (everything from airplanes to toothbrushes). Engineers make new products and keep them working.

Answer choices: Not at all interested (1), Not so interested (2.33), Interested (3.66), Very interested (5)

**Math Potential:**

Question prompt: In the future, I could do harder math problems.

Answer choices: Strongly Disagree (1), Disagree (2), Neither Agree nor Disagree (3), Agree (4), Strongly Agree (5)

**Coding Experience:**

Question prompt: How would you describe your experience with coding or computer programming (in any language)?

Answer choices: I have never coded before (1), I’ve tried coding a little (e.g., Hour of Code, online game or app) (2.33), I’ve written short programs before (e.g., in Python, Java, Scratch, etc.) (3.66), I have taken a class or done a full project in coding (5)

**Coding Confidence:**

Question prompt: How confident do you feel about writing simple code (e.g., using variables or functions)? Scale from 1 (Not at all confident) to 5 (Very confident).

Answer choices: 1 (1), 2 (2), 3 (3), 4 (4), 5 (5)

**Coding Perception:**

Question prompt: Do you think coding can be used to explore or solve problems in science, like materials science?

Answer choices: Definitely not (1), Probably not (2), Not sure (3), Probably yes (4), Definitely yes (5)

## References

(1) *The structure and properties of chocolate*. RSC Education. https://edu.rsc.org/experiments/the-structure-and-properties-of-chocolate/688.article (accessed 2025-09-30).

(2) *How Strong is Your Chocolate Lesson - Ceramic and Glass Industry Foundation*. https://foundation.ceramics.org/. https://foundation.ceramics.org/teacher-resources/free-lesson-plans/strong-chocolate/ (accessed 2025-09-30).

(3) Yin, D.; Shumeyko, C. M.; Cline, J. E.; Dunstan, M. K.; Goins, P. E.; Field, D. M. Snap, Crackle, and Pop: Breaking Chocolate to Understand Composite Design. *J. Mater. Educ.* **2019**, *41* (1–2), 27–40.

(4) *Investigating Materials Using Chocolate*. https://www.materials.ox.ac.uk/workshops-schools (accessed 2025-09-30).

(5) *Student Attitudes toward STEM Survey (S-STEM): Middle and High School (6–12th)*; Friday Institute for Educational Innovation: Raleigh, NC, 2012. https://csedresearch.org/wp-content/uploads/Instruments/STEM/PDF/MISO_S-STEM_MiddleHigh_09-20-12_PUBLIC.pdf (accessed Sept 30, 2025).

## Appendix 1. Handout for Building Models of Triglycerides


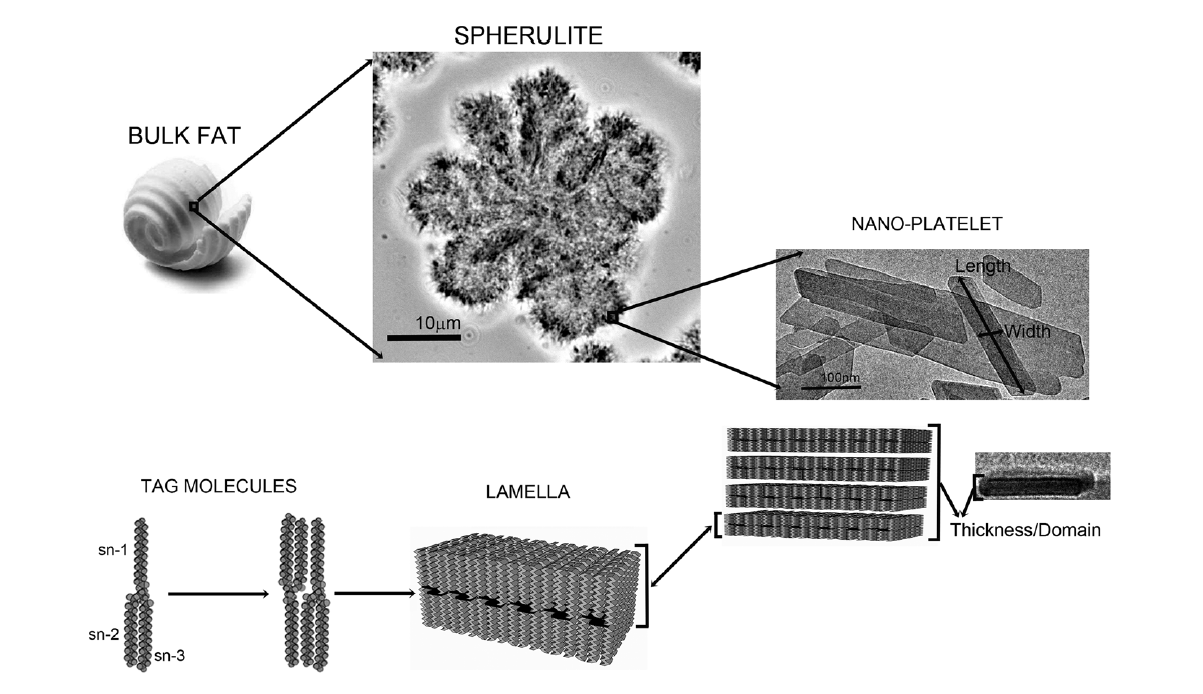


Image reproduced from *Soft Matter*, 2012, 8, 1275–1300

**Part 1. Exploring a fat molecular structure**


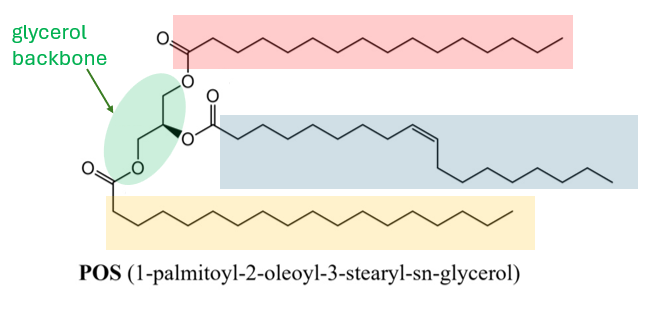
Triglycerides are a type of fat molecule. They are also known as triacylglycerols (TAGs) because they are composed of a glycerol molecule and three fatty acids linked by ester bonds.

A representative TAG is shown below with the major sections highlighted: (1) the glycerol backbone is highlighted green, and the three fatty acid chains are highlighted red, blue, and yellow. Often, TAGs are represented by stick illustrations, as shown in Figure 2. There are four different configurations that the TAG can adopt: “E”, tuning fork, chair, and propeller.

**Figure 1.** Structure of a triacylglycerol TAG. Adapted from Reproduced from *Crystal Growth & Design* **2025** *25*, 2764-2783.


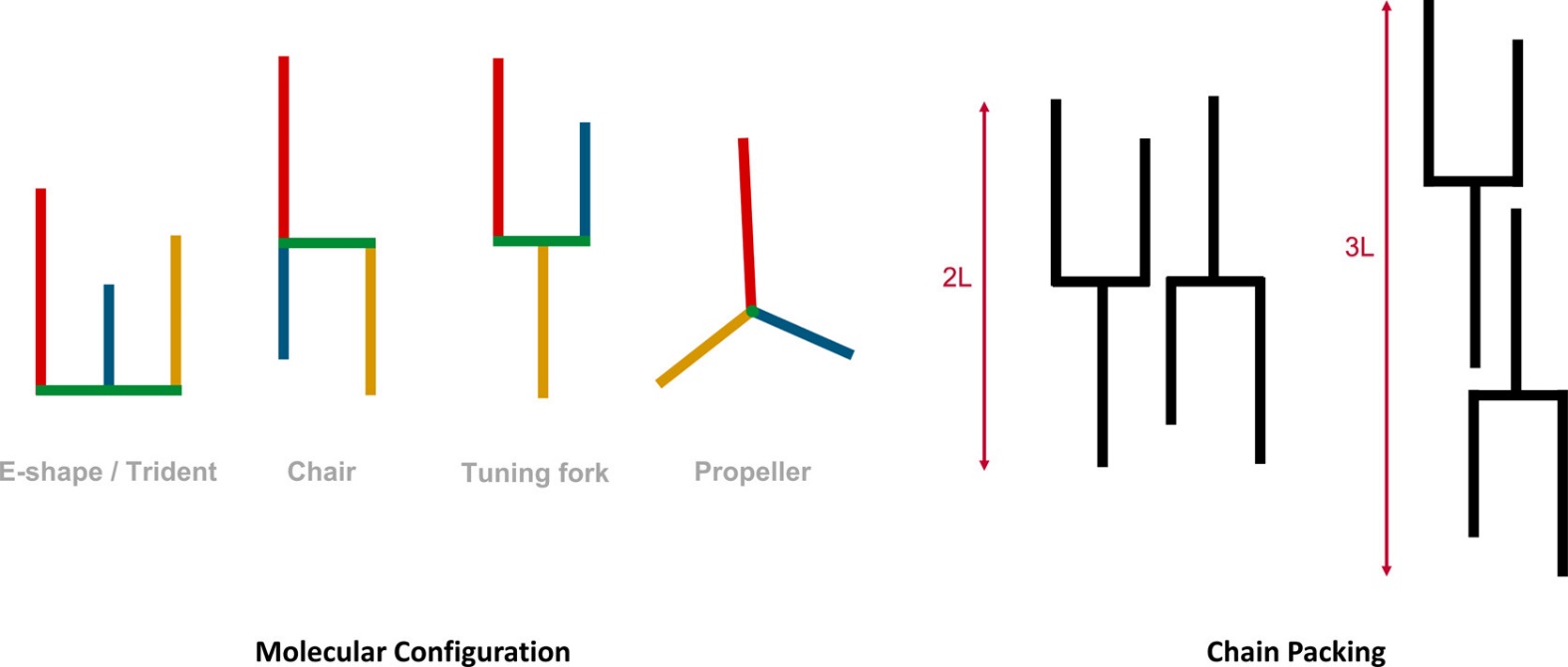


**Figure 2.** Illustrations representing the different TAG configurations. Reproduced from *Crystal Growth & Design* **2025** *25*, 2764-2783.

In your kit, you will find the following components

- three connectors
- two short straws, same color
- three longer straws, each a different color

Use these components to build a structure that represents the tag molecule in the “E” configuration. Take a picture with your laptop camera.

Without disconnecting any of the components, rotate the structure about the connectors to change it to the tuning fork and chair configurations.

Return the structure to the “E” configuration and hold the structure so that it is on its edge, and you see one chain (with the other two chains blocked from your view behind the first chain). Rotate the connectors to achieve the “propeller configuration”.

Open Google Slides, and insert the pictures of the different TAG configurations. Label each configuration.

**Part 2. Assembling fat molecules**


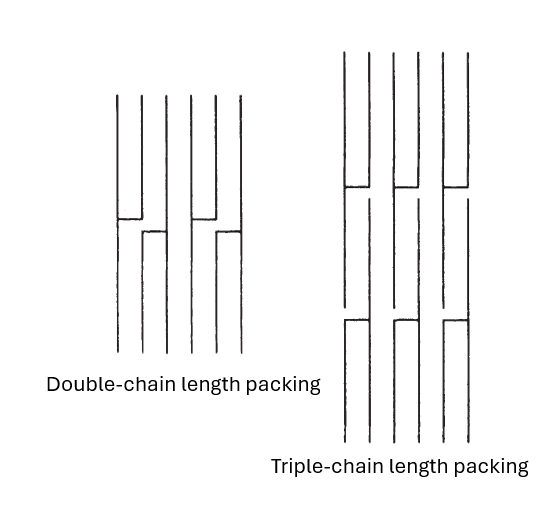
When fats crystallize, the molecules pack in an ordered arrangement, usually with the chair or tuning fork configuration. For this exercise, we will consider the chair configuration.

The chains can arrange either in a bilayer (two layers of chains) or a trilayer (three layers of chains), as shown in Figure 3.

In your kit, you will find connectors, short and longer straws, all the same color.

**
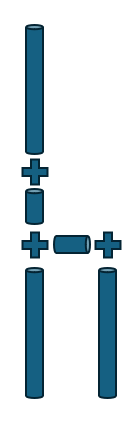
(1)** Start by building a single chair molecule as shown in Figure 4. This is a different construction from part 1, only to simplify the construction of the chair. Build at least 9 of these molecules.

**Figure 3.** Typical packing arrangements of chair TAG molecules. Adapted from Talbot (1995). Fat eutectics and crystallisation.

**(2)** Work with your classmates to assemble the molecules into a trilayer of chains consisting of 12 molecules, where adjacent molecules are different colors. First arrange and order all the molecules on a flat surface. Then, to hold the molecules together, use the transparent tubes (extra connectors may be needed to hold the layer together). Take a picture of your layer and insert into the Google Slides report.

Build at least one additional layer of 12 molecules.

When you stack your layers of fat molecules, you have formed a lamella!

**Figure 4.** Strategy for constructing a chair molecule for part 2.

arrangements of chair TAG molecules

## Appendix 2. Handout for Investigating the melting behavior of chocolate

This procedure is reproduced, with slight modification, from RSC Education:

<https://edu.rsc.org/experiments/the-structure-and-properties-of-chocolate/688.article>

A key ingredient of chocolate is cocoa butter. It is a fat with at least six different crystalline forms. This means that the atoms are the same, but they are arranged in a different way. The different arrangements can lead to varying properties, including melting point, ease of snapping, strength, gloss, and texture.

The ability of the structure to take on many different crystalline forms is called polymorphism. (‘poly’ means many; morph’ means shape). Chocolate polymorphism is a complex phenomenon, and it remains an area of active research. Cocoa butter has six polymorphs (Table 1). The form V polymorph has a far superior taste and texture than the others. It is also the glossiest and snaps well.

**Table 1. Characteristics of different cocoa butter polymorphs.**

| **Cocoa butter polymorph** | **Conditions to make the polymorph** | **Melting point (°C)** |
| --- | --- | --- |
| Form I | Rapidly cooling molten chocolate | 17.3 |
| Form II | Cooling the molten chocolate at 2 °C | 23.3 |
| Form III | Solidifying the molten chocolate at 5-10 °C (or storing ‘form II’ at 5-10°C) | 25.5 |
| Form IV | Solidifying the molten chocolate at 16-21 °C (or storing ‘form III’ at 16-21 °C) | 27.3 |
| Form V | Solidifying the molten chocolate whilst stirring. Needs a special process called ‘tempering’ | 33.8 |
| Form VI | Storing ‘form V’ at room temperature for four months | 36.3 |

**Melting point tests**

1. Use a double boiler or a test tube in a beaker for this experiment. Pour hot water from a kettle (approximately 55˚C) into the outer container, i.e., our the water bath.
2. Place a few small pieces of chocolate (enough to cover the tip of a thermometer when melted) into the inner container, and put in the thermometer.
3. Take the temperature of the chocolate, then put the inner container in the water bath and start the timer.
4. Stir continuously with the thermometer and record the temperature of the chocolate every 10 seconds for about two minutes or until the temperature stops changing. Write down any observations.
5. Repeat steps 1–4 with chocolate that has been melted and rehardened.
6. Using Excel or Google Sheets, plot a graph of each set of results and use them to decide the melting point of the samples and if the samples have the same structure. Use Table 1 to suggest which form of cocoa butter might be in your samples.
7. Bring your samples to the DSC for testing.

## Appendix 3. Handout for Mechanical properties of chocolate

**Part 1: How strong is your chocolate**

Procedure adapted from https://ceramics.org/wp-content/uploads/2014/04/How-Strong-is-your-Chocolate-Lesson.pdf

**Introduction:** Materials such as *metals* (aluminum, iron, copper, etc.), *ceramics* (porcelain, silicon carbide, etc.) and *polymers* (milk jugs made of polyethylene) are tested by scientists and engineers to reveal the material’s mechanical properties. One type of mechanical testing is strength testing. Strength is a measurement of the maximum stress that a material can withstand. Many of the materials that we see every day are subjected to a variety of stresses and must be designed to provide a certain measure of strength.

The atomic structure of a material is a major factor that influences the strength of a material and involves the elements in the material – the way they are bonded to each other and the way the atoms are arranged to make different structures. However, two materials that share all of the same atomic traits can still have different strengths if their microstructure is altered due to processing. The chocolate bars in this lab are an excellent example of how microstructure can be altered due to processing. The chocolate in all of the bars has the same elemental make-up and atomic traits. However, the microstructures differ due to things that have been added to the chocolate, such as almonds.

**Lab Description:** In this lab, you will measure the strength of a chocolate bar. The flexural strength of the chocolate bars will be measured using a conventional 3-point bending test set-up (see Figure 1). For this test set-up, chocolate bars are placed on two supports (making two points of contact), and a force is applied to the center of the bar (making the 3rd point of contact in a 3-point bending test).

***Figure 1. Test set-up for a 3-point bending test***

**Instructions:**

1. Measure and record on your data sheet the following information about the bar:
   1. Type (milk chocolate, dark chocolate, almond, etc.)
   2. Width of the bar (mm), *w*
   3. Thickness of the bar (mm), *t*
2. Position two desks so that the chocolate bar can span across the space between the desks. Approximately ½ inch of the chocolate bar should be touching each desk.
3. Measure and record (in mm) on your data sheet the length of the chocolate bar that is not supported by the desks. This is called the length of the support span, *L*.
4. Place the twine with the cup attached across the middle of the chocolate bar so that the cup hangs freely below the chocolate bar as shown in Figure 2.

***Figure 2. Chocolate bar subjected to a 3-point bending test***

1. Place a container under the chocolate to catch everything when it falls.
2. Create a paper funnel by rolling a piece of paper and taping it.
3. Using the funnel, start placing the weights into the cup, one at a time. The weights can be pennies, marbles, washers, etc. The weights should be funneled in at a steady pace, ensuring that each weight lands in the cup before the next one enters the cup. Try funneling the weights in a way that they do not fall a large distance when they enter the cup.
4. Continue placing weights into the cup at a steady rate until the chocolate bar fractures. Be sure to note any deflections or bending of the chocolate bar during the loading process. ***NOTE:*** If it is difficult to see the bar start to deflect, place the ruler across the desk just to the side of the chocolate bar to help indicate when the bar starts to deflect from a horizontal line.
5. Look at the fracture surface and record any observations.
6. Find the mass (in grams) of the cup, string, and the weights in the cup at fracture using a balance. The force, *P*, applied to the chocolate bar can then be calculated as follows:

*P* = (mass of cup, twine, and weights)*(acceleration due to gravity = 9.81m/s^2^)

1. Use the force, *P*, to calculate the flexural strength of the chocolate bar. The formula for calculating flexural strength is:

$$\sigma=\frac{1.5PL}{wt^{2}}$$

where σ is the flexural strength (MPa), *P* is the applied force (N), *L* is the length of the

support span (mm), *w* is the width of the bar (mm), and *t* is the thickness of the bar (mm).

**Data sheet for part 1**

| **Type of chocolate bar** | **Width, *w*** | **Thickness, *t*** | **Length of support span, *L*** |
| --- | --- | --- | --- |
|  |  |  |  |
| Changes in the bar during the loading process: | | | |
| Observations of the fracture surface: | | | |
| Mass of the cup/twine/weights: | | | |
| Calculation of load, *P*: | | | |
| Calculation of the bar’s flexural strength, *σ*: | | | |

**Part 2. How hard is your chocolate?**

Procedure adapted from

<https://pspb.org/nano/media/Hardness_Choc_MS_Lab_v102doc.pdf>

<https://www.materials.ox.ac.uk/files/makingmaterialsmatter2017-chocolatepdf>

Hardness is probably a concept you are well familiar with. You already know that certain materials are harder than others; in fact, you prove it every day when you chew your food and your teeth don’t break (because your teeth are harder than the foods you chew). **Hardness can be defined as a material’s ability to resist a change in shape.** In 1812, Friedrich Mohs came up with a way of ranking materials on a comparative scale – he simply took 2 different materials and observed which one became scratched when they were rubbed together. Since then, a more quantitative measure of hardness has been developed. Modern hardness testers take a well-defined shape and press it into a material with a certain force, observing the indent it leaves in the material when it is removed.

Today, you will be performing hardness testing on chocolate bars.

While there are many types of indenter heads used in measuring hardness, one of the common ones is simply a hard sphere. We will be able to mimic this indenter tip using a metal sphere.

**Instructions:**

Note: during the experiment, be sure to record all observations (i.e., surface smoothness, any cracking in the chocolate after an indent, etc.)

1. Measure the diameter of your metal sphere indenter with the calipers. You may wish to take several measurements and average them for added accuracy.
2. Measure the mass of your indenter.
3. Roll up a sheet of paper and tape it in place so it creates a hollow tube that would allow your indenter to fall through. This will serve to make sure your indenter will fall straight down.
4. Unwrap one of your chocolate bars and set it on its wrapper face down, so that the flatter surface sticks up (lettering or grooves facing down). Make observations of your chocolate bar (notice cracks, indents, nuts, etc.)
5. Measure the height of your sheet of paper. This height is important because it will be the height that your indenter falls and will determine the force of your indentation.
6. Place the rolled paper on top of the chocolate. Hold the indenter so that it just barely hovers over the opening of the paper. DROP YOUR INDENTER. After it has fallen and impacted your chocolate, carefully remove it.
7. Measure the diameter of the indent it left behind. If the indent is not visible, try dropping it from a taller height, but make sure to record these modifications. Be sure to write down any relevant observations.
8. You may wish to indent each chocolate bar more than once. If you do, be sure to space the indents appropriately. You can get more than 10 indents per bar!
9. Make observations of your chocolate bar after it has been indented.
10. Use a larger piece of paper (or multiple sheets of paper taped together) to test a fall from a different height. Measure the height of your sheet of paper. This height is important because it will be the height that your indenter falls and will determine the force of your indentation.
11. Repeat the procedure to determine the force of indentation from your new height. In all cases, be sure to record both the height of the drop and the diameter of the indentation.

## Appendix 4. Handout for the Polarized light kit activity

***Prof. Noel C. Giebink of the University of Michigan designed this activity for PUMA 2025.***

For this activity, you will need:

1. An LED tap light
2. An adhesive paper label (diffuser)
3. An adhesive-backed linear polarizing film (orange tape) with protective layers on both sides
4. A non-adhesive polarizing film (blue tape) with protective layers on both sides
5. A magnifying glass
6. Linear polarized glasses with paper frames


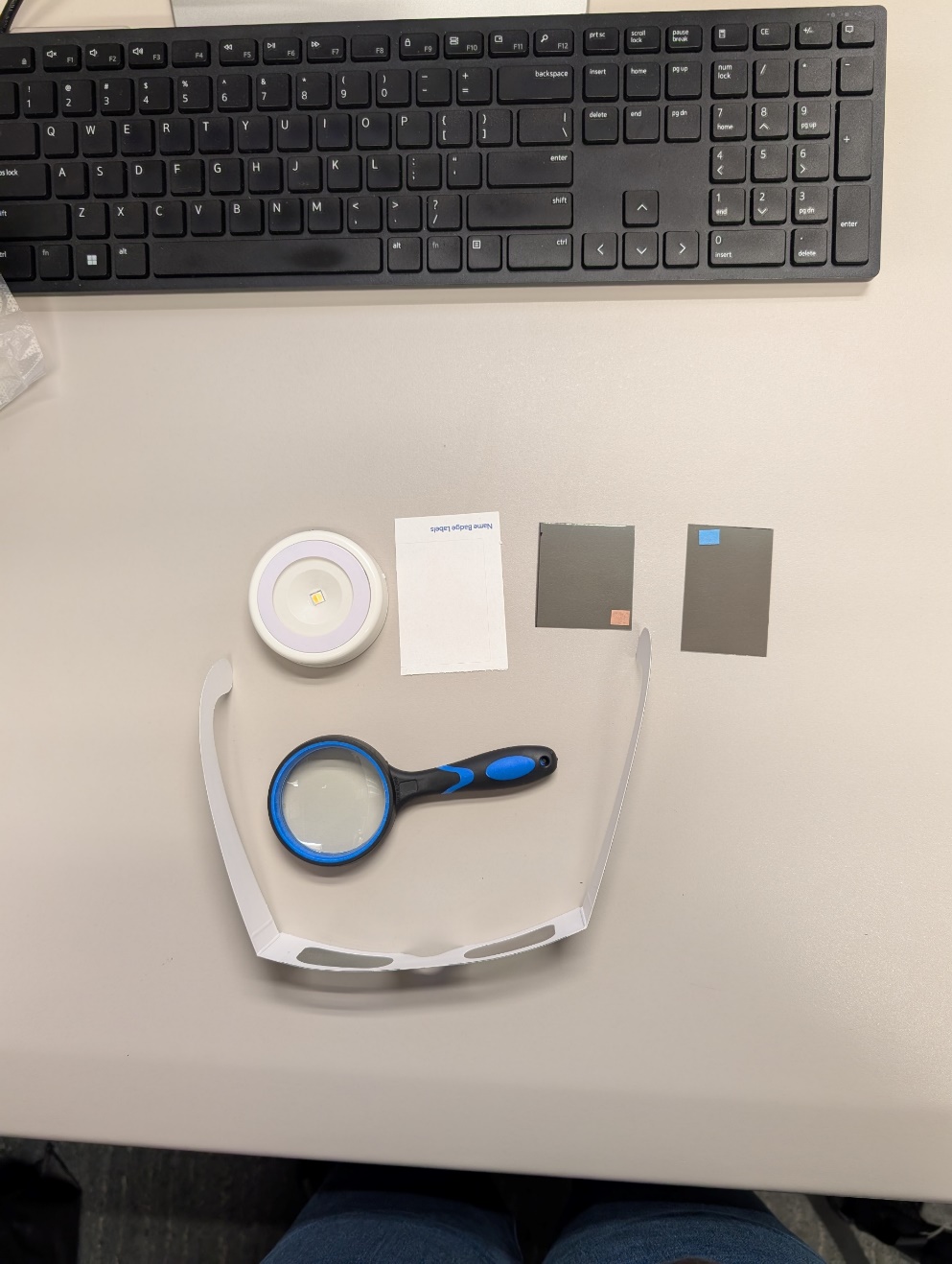


1

2

3

4

5

6

**Figure 1. Contents of the crossed polarizer light kit**

**1. Instructions for making a linearly polarized backlight:**

1. Take the adhesive-backed linear polarizing film (orange tape) and remove the protective layer from the adhesive side (the side with the orange tape).
2. Stick the polarizing film to the paper diffuser, pressing firmly to remove any bubbles
3. Cut the diffuser-polarizer so that it covers the illuminated area of the light: either cut a square with sides = 4 cm or a circle with diameter = 4 cm
4. Remove the backing from the paper side, and stick the diffuser-polarizer to the light surface, pressing firmly over the entire area.
5. Remove the protective film from the top of the polarizer

**2. Operating the linearly polarized backlight**

1. The light has an ON/OFF switch on the side
2. To change the color temperature, double-tap the surface of the light
3. To adjust the brightness of the light, press and hold the light surface.
4. For the polarizer, use the brightest setting.
5. The LED can be charged with a USB-C charger

**3. Observing objects between crossed polarizers**

1. Turn on the tap light
2. Hold an object with one hand in front of the polarized backlight (or lay it gently on the surface of the backlight)
3. Put on the polarized glasses. Rotate the polarized light and observe the object. What do you see?
4. You can use the magnifying glass to look more closely at your object through the crossed polarizers
5. Instead of the polarized glasses, a second non-adhesive polarizing film is provided to place on top of the object, such as a microscope slide sample
